# A Natural Metabolite and Inhibitor of the NLRP3 Inflammasome: 4-hydroxynonenal

**DOI:** 10.33696/immunology.6.192

**Published:** 2024

**Authors:** Jinmin Zhang, Bradford C. Berk, Chia George Hsu

**Affiliations:** 1 Department of Pharmaceutical Sciences, College of Pharmacy, University of North Texas Health Science Center, Fort Worth, TX, USA; 2 Department of Medicine, Aab Cardiovascular Research Institute, University of Rochester School of Medicine and Dentistry, Rochester, NY, USA; 3 Department of Physical Medicine and Rehabilitation, University of Rochester School of Medicine and Dentistry, Rochester, NY, USA; 4 Department of Kinesiology, The University of Texas at San Antonio, San Antonio, TX, USA

**Keywords:** NLRP3, 4-hydroxynonenal, Inflammation, Macrophage, Pyroptosis

## Abstract

The NOD-, LRR-, and pyrin domain-containing protein 3 (NLRP3) inflammasome, crucial in the innate immune response, is linked to various human diseases. However, the effect of endogenous metabolites, like 4-hydroxynonenal (HNE), on NLRP3 inflammasome activity remains underexplored. Recent research highlights HNE’s inhibitory role in NLRP3 inflammasome activation, shedding light on its potential as an endogenous regulator of inflammatory responses. Studies demonstrate that HNE blocks NLRP3 inflammasome-mediated pyroptosis and IL-1β secretion. Additionally, covalent targeting emerges as a common mechanism for inhibiting NLRP3 inflammasome assembly, offering promising avenues for therapeutic intervention. Further investigation is needed to understand the impact of endogenous HNE on NLRP3 inflammasome activation, especially in settings where lipid peroxidation byproducts like HNE are produced. Understanding the intricate interplay between HNE and the NLRP3 inflammasome holds significant potential for unraveling novel therapeutic strategies for inflammatory disorders.

## NLRP3 Inflammasome and Human Diseases

The NLRP3 inflammasome is one of the most extensively studied inflammasomes, implicated in various human diseases such as rheumatoid arthritis [1], Alzheimer’s disease [2], Type 2 diabetes [3], sepsis, as well as tissue injuries like acute lung injury and spinal cord injury [4–6]. This multiprotein complex plays a crucial role in the innate immune response, comprising proteins such as NLRP3, apoptosis-associated speck-like protein containing a caspase activation and recruitment domain (CARD) (ASC), and pro-caspase-1. NLRP3 inflammasome activation occurs within host cells in response to pathogen-associated molecular patterns (PAMPs) and damage-associated molecular patterns (DAMPs) [7,8]. During this process, inactive interleukin-1β precursor (pro-IL-1β) and interleukin-18 precursor (pro-IL-18) are cleaved to mature inflammatory cytokines interleukin-1β (IL-1β) and interleukin-18 (IL-18), which are then released into the intercellular space through gasdermin membrane pores [3,9–12]. Despite the well-defined mechanisms of inflammasome activation, the role of endogenous metabolites in resolving inflammasome activation remains underappreciated.

## Biology of 4-hydroxynonenal

Lipid mediators play crucial roles in regulating inflammation. Among reactive aldehydes derived from lipid peroxidation, 4-hydroxynonenal (HNE) stands out as the most abundant end-product [13,14]. Under physiological conditions, the concentration of HNE in human serum ranges from 0.05 to 0.15 μM [15]. However, HNE levels may increase to 3–6 μM in tissues under oxidative stress [16,17]. Glutathione peroxidases (GPXs) are antioxidant enzymes crucial for protecting cells from lipid peroxidation [18]. Notably, deficiency or inhibition of glutathione peroxidase-4 (GPX4) enhances the production of endogenous HNE both *in vivo* and *in vitro* [19–21]. HNE has emerged as a significant second messenger signaling molecule due to its unique chemical structure, which includes an aldehyde functional group (-CHO) positioned at the fourth carbon atom of a nine-carbon chain. This structure renders HNE relatively reactive and prone to reacting with protein residues [13,22]. In this review, we will explore the inhibitory effects of HNE on NLRP3-mediated pyroptosis in macrophages, exploring its impact on Nrf2 activation, NF-κB signaling, and the NEK7-NLRP3 interaction in macrophages. Our goal is to deepen our understanding of the mechanisms underlying HNE’s inhibition of NLRP3 inflammasome and to advance our knowledge in this area.

## NLRP3 Inflammasome Activation

The stimulation of the NLRP3 inflammasome involves two signals: 1) Priming, which entails the transcription and translation of inflammasome components; and 2) Activation, which leads to inflammasome assembly [23]. Initially, priming enhances the transcription and translation of NLRP3 by activating TLR4 and subsequent NF-κB signaling [24–26]. Subsequently, activation of NLRP3 inflammasome assembly is triggered by a cell danger signal, such as K^+^ efflux, or extracellular ATP for NLRP3 [23]. Post-translational modifications (PTMs) in NLRP3 during the second step promote inflammasome assembly. These PTMs encompass deubiquitination, phosphorylation, nitrosylation, acetylation, alkylation, and ADP-ribosylation [27–31]. Therefore, PTMs in NLRP3 are pivotal for its inflammasome assembly. In contrast to the NLRP3 inflammasome, NLR family CARD domain containing protein 4 (NLRC4) inflammasome assembly can be activated by flagellin [32,33], and the pyrin and HIN domain containing protein family absent in melanoma 2 (AIM2) inflammasome can recognize cytosolic double-stranded DNA (dsDNA) through its DNA-binding HIN domain, initiating inflammasome assembly [34]. During activation, the NLRP3 inflammasome assembly necessitates an essential modulator called NIMA-related kinase 7 (NEK7). NEK7 directly binds to NLRP3 to bridge adjacent NLRP3 subunits and regulate oligomerization [35,36]. It is important to note that in the absence of NEK7, caspase-1 activation and IL-1β release were halted in response to signals that activate NLRP3 but not NLRC4 or AIM2 inflammasomes [35].

## Effect of HNE on NLRP3 Inflammasome Activation

Recently, Hsu *et al*. discovered that HNE blocks NLRP3 inflammasome-mediated pyroptosis and the secretion of IL-1β by mouse primary macrophages and human peripheral blood mononuclear cells [37]. They demonstrated that treatment with HNE, or an increase in endogenous HNE levels, led to a reduction in inflammasome activation in mouse models of acute lung injury and sepsis [37]. These findings establish HNE as a novel endogenous inhibitor of the NLRP3 inflammasome, shedding light on the role of endogenous lipids in modulating inflammatory responses associated with pyroptosis. Nuclear factor erythroid 2-related factor 2 (Nrf2) has been proposed to decrease cellular reactive oxygen species (ROS), repress proinflammatory gene transcription, and inhibit NLRP3 inflammasome activation in macrophages [38–41]. This mechanism was supported by evidence showing that electrophiles (such as sulforaphane, tert-butyl hydroquinone, dimethyl fumarate, and itaconate) exhibited anti-inflammatory responses through Nrf2 pathways [41–45]. To determine if Nrf2 activation was necessary for HNE inhibition of pyroptosis, Hsu *et al*. blocked Nrf2 signaling using the specific inhibitor ML385. They found that HNE still prevented cell death in the presence of ML385, suggesting that the protective effect of HNE against pyroptosis was independent of the Nrf2 pathway [37]. Inflammasome activation involves priming-dependent stimulation of NLRP3 expression, followed by NLRP3 assembly promoted by a cell danger signal, such as K^+^ efflux or ATP. To minimize the effects of HNE on inflammasome priming, macrophages were treated with LPS for 3 hours before exposure to HNE and then stimulated with nigericin or ATP [37]. Hsu *et al*. observed that HNE completely prevented IL-1β maturation, indicating that the inhibitory effect of HNE treatment on inflammasome activation is independent of the priming pathway [37]. To examine the effect of HNE on inflammasomes in macrophages, Hsu *et al*. employed LPS priming and induced K^+^ efflux via nigericin, ATP, or imiquimod as secondary activation signals to trigger the NLRP3 inflammasome [37]. They utilized cytosolic dsDNA poly(dA:dT) to activate the AIM2 inflammasome, and cytosolic flagellin to activate the NLRC4 inflammasome [37]. Their findings indicated that HNE specifically reduced cleavage of caspase-1 and IL-1β release mediated by the NLRP3 inflammasome, rather than the AIM2 and NLRC4 inflammasomes. Given that NLRP3, NLRC4, and AIM2 inflammasomes share common downstream effectors such as ASC and gasdermin D (GSDMD) for inducing pyroptosis [8,46], these results suggest the selectivity of HNE towards inhibiting NLRP3 inflammasome activation.

## Effect of HNE on NLRP3 – NEK7 Interaction

Regarding the mechanism of NLRP3 inflammasome activation, Hsu *et al*. discovered that HNE directly bound to the NLRP3 sensor in a cysteine-dependent manner [37]. They employed several methods, including alkyne-HNE and azido click-chemistry method, as well as the coincubation of HNE and N-acetylcysteine (NAC), to verify this binding. To detect the physical association between NEK7 and NLRP3, co-immunoprecipitation assays were conducted. It was observed that NEK7 was linked with NLRP3 post LPS/nigericin treatment, and this association was inhibited by HNE treatment [37]. These findings demonstrated that HNE mitigated NLRP3 inflammasome activation by blocking the interaction between NLRP3 and NEK7 during the formation of NLRP3 inflammasome.

## Future Perspectives

1) Covalent targeting has been shown as a common mechanism for inhibiting NLRP3 inflammasome assembly (Table 1). Besides HNE, there are many other molecules that can also inhibit NLRP3 inflammasome activation through cystine modification on NLRP3, thereby blocking NEK7-NLRP3 interaction. For example, another electrophilic metabolite, itaconate, and its derivative, 4-Octyl itaconate (4-OI), can also inhibit NLRP3 inflammasome activation by dicarboxypropylating cysteine 548 on NLRP3 [47]. Oridonin, a diterpenoid compound isolated from the Chinese medicinal herb *Rabdosia rubescens*, functions as an NLRP3 inflammasome inhibitor. Its mechanism involves the specific targeting of cysteine 279 within the NLRP3 protein [48]. Regarding other electrophiles, CY-09 directly binds to the ATP-binding motif of the NLRP3 NACHT domain, leading to the inhibition of NLRP3 ATPase activity. This inhibition results in the suppression of NLRP3 inflammasome assembly and activation [49]. Similarly, OLT1177, an orally active beta-sulfonyl nitrile, acts as a direct inhibitor of the ATPase activity of the NACHT domain of NLRP3, thereby preventing NLRP3 oligomerization [50]. In terms of drugs approved by the U.S. Food and Drug Administration (FDA), Tecfidera is used for treating relapsing multiple sclerosis, containing dimethyl fumarate (DMF) as its active ingredient. Studies suggest that DMF inhibits macrophage pyroptosis by succinating GSDMD at human/mouse Cys191/Cys192 [51]. Similarly, disulfiram, approved by the FDA for treating chronic alcoholism, covalently modifies Cys191/Cys192 in GSDMD [52]. Given that GSDMD is implicated in pyroptotic pore formation, blocking GSDMD cleavage or pore formation not only prevents pyroptosis but also inhibits the release of IL-1β and IL-18 into the extracellular space.

2) Emerging research demonstrates that electrophiles like HNE and itaconate activate Nrf2 by modifying cysteine residues on Kelch-like ECH-associated protein 1 (Keap1), thereby preventing Nrf2 degradation and promoting its translocation into the nucleus [37,41]. This activation leads to increased expression of Nrf2 target genes such as glutamate-cysteine ligase catalytic subunit (GCLC) and ferroportin-1, highlighting Nrf2 activation as a critical mechanism mediating the cytoprotective effects of HNE during oxidative stress [37]. Given the dual effects of HNE on Nrf2 activation and inhibition of the NLRP3 inflammasome, it is plausible that HNE contributes to the resolution of inflammation following infections or tissue injuries. Specifically, during infection, macrophages rapidly accumulate in injured tissues and produce reactive oxygen species to combat pathogens. These activated macrophages can persist at the injury site for prolonged periods, potentially leading to excessive inflammation and tissue damage [53]. Elevated levels of HNE during lipid peroxidation may suppress the NLRP3 inflammasome and activate Nrf2 through its electrophilic nature, thereby modulating inflammatory responses and supporting tissue repair during lipid peroxidation observed in viral infections or tissue injuries. The role of HNE in tissue damage may facilitate macrophage phenotype transition, resolution of inflammatory processes, and contribute to tissue healing and recovery.

3) Several FDA-approved nonspecific anti-inflammatory drugs have demonstrated effectiveness in inhibiting inflammatory reactions. For instance, phosphodiesterase 4 (PDE4) inhibitors like Rolipram and roflumilast are FDA-approved to treat chronic obstructive pulmonary disease (COPD) and psoriasis [54]. Studies have shown that PDE4 inhibitors block the NLRP3 inflammasome by increasing cyclic adenosine monophosphate (cAMP) levels through the PKA pathway and by directly interacting with cAMP-NLRP3 [54,55]. Despite the success of NLRP3 blockers in preventing or treating numerous preclinical animal disease models, and with some NLRP3 inhibitors entering early clinical trials, there are currently no FDA-approved NLRP3 inhibitors. Instead of administering HNE directly in vivo to inhibit inflammation, a promising strategy involves increasing HNE production by inhibiting the HNE metabolic pathway in macrophages. Specifically, enzymes like glutathione S-transferases (GSTs) and glutathione peroxidase (GPX) facilitate the addition of glutathione (GSH) to HNE [19,22]. Blocking macrophage-specific GSTs or GPX could boost HNE production within macrophages, thereby suppressing the inflammatory response [19,56]. Advancing our understanding of HNE metabolism will offer valuable insights into how lipid metabolism influences macrophage function and could reveal new clinical strategies to promote inflammation resolution.

4) The role of endogenous HNE on NLRP3 inflammasome. Ozone is a highly reactive gas that is found in the Earth’s atmosphere, particularly in areas with high levels of air pollution. Inhalation of ozone can lead to oxidative stress in the respiratory tract, resulting in lipid peroxidation and the production of HNE [57]. Continuous ozone exposure may inhibit NLRP3 inflammasome. Exercise-induced oxidative stress can lead to the generation of HNE, which has been implicated in regulating inflammation [58]. However, the exact mechanisms by which exercise-induced HNE production may influence the NLRP3 inflammasome are not fully understood and may vary depending on factors such as the intensity and duration of exercise. Further research is needed to elucidate the specific effects of exercise-induced HNE on the NLRP3 inflammasome and its implications for inflammatory processes in the body.

Overall, HNE demonstrates potential in inhibiting inflammation through multiple mechanisms, as outlined in our review. A mechanistic link between these observations emerges in the intracellular binding of HNE to precise cysteine residues, facilitating oxidative assistance and subsequent formation of intra-molecular bonds. Much like the narrative of Goldilocks and the Three Bears, the levels of endogenous HNE dynamically fluctuate, finely shaping cellular outcomes. Further research is warranted to fully understand the specific effects of endogenous HNE on the NLRP3 inflammasome and its broader implications for inflammatory processes in the body.

## Figures and Tables

**Table 1.** Covalent targeting NLRP3 inflammasome pathways.

Electrophiles	Mian Target	IL-1β inhibition % (μM)	References
4-hydroxynonenal	Cystine dependent NLRP3	100% (3 μM)	37
4-Octyl itaconate (4-OI)	Cystine 548 of NLRP3	100% (500 μM)	47
Oridonin	Cysteine 279 of NLRP3	100% (2 μM)	48
CY-09	NLRP3 ATPase	100% (10 μM)	49
OLT1177	NLRP3 ATPase	60% (1 μM)	50
Dimethyl Fumarate	Cysteine 191/192 of GSDMD	100% (25 μM)	51
Disulfiram	Cysteine 191/192 of GSDMD	100% (40 μM)	52

a. IL-1β inhibition % (μM) indicates the concentration required for electrophile to inhibit indicated percentage of IL-1β release after NLRP3 inflammasome activation.
